# Down-regulated aquaporin 5 inhibits proliferation and migration of human epithelial ovarian cancer 3AO cells

**DOI:** 10.1186/s13048-014-0078-2

**Published:** 2014-08-15

**Authors:** ChunXiao Yan, Yunshan Zhu, Xiao Zhang, Xuejun Chen, Wei Zheng, Jianhua Yang

**Affiliations:** Department of Gynecology, The Second Affiliated Hospital, School of Medicine, Zhejiang University, Hangzhou, Zhejiang Province China; Department of Obstetrics & Gynecology, Sir Run Run Shaw Hospital affiliated to the Zhejiang University School of Medicine, Hangzhou, Zhejiang Province 310016 China

**Keywords:** 3AO cell, Aquaporins 5, Migration, Proliferation, Tumorigenesis

## Abstract

**Background:**

Recent studies suggested that aquaporins 5 (AQP5) was associated with many kinds of cancers and regulated many processes of various kinds of cancer cells. Our previous studies also demonstrated that AQP5 was highly expressed in epithelial ovarian cancer and contributed to the progress of ovarian cancer.

**Methods:**

Lentivirus for knocking-down the expression of AQP5 was prepared and verified by qPCR and Western blotting. Cell counting kit-8 (CCK8) assay and transwell assay were performed to investigate the role of AQP5 on proliferation and migration of 3AO cells. The effects of down-regulating AQP5 on tumorigenesis were tested by tumor xenografts experiments.

**Results:**

An effective lentivirus silencing AQP5 expression was obtained and used in this study. Down-regulating AQP5 inhibited proliferation and migration of cultured human epithelial ovarian cancer 3AO Cell. Furthermore, interfering of AQP5 during tumorigenesis could efficiently decrease the tumor growth in athymic mice.

**Conclusions:**

These findings altogether suggest that AQP5 regulated multi processes in ovarian carcinogenesis and may be an attractive therapeutic target.

## Background

Ovarian cancer is a very serious cancer. It is estimated that there will be 21,980 ovarian cancer cases newly diagnosed and 14,270 deaths due to ovarian cancer among female in the United States in 2014 which suggested that ovarian cancer will be the eleventh common cancer and the fifth common cause of cancer death in women [1]. Early stage ovarian cancer has a good 5 year survival rate at approx. 92%, although <30% of patients will present with early stage disease. The majority of ovarian cancers present at late stage disease (5 year survival 30%) as the clinical symptoms of ovarian cancer in the early stages are vague [2,3].Tumor-debulking surgery combined with following plantinum-taxane combination chemotherapy has been the fist-line treatment for ovarian cancer for over twenty year with very high response rate in the initial stage of therapy, while most patients with advanced-stage ovarian cancer relapse within 18 months and finally die from the disease [4].

Aquaporins (AQPs) are a family of small integral plasma membrane proteins expressed in all living organisms [5]. AQPs primarily transport water and in some cases, a wide range of nonpolar solutes such as urea and glycerol and even some unconventional molecules such as the nonpolar gases carbon dioxide and nitric oxide [6]. In humans, there are 13 kinds of Aquaporins members divided into three subgroups as water selective AQPs, aquaglyceroporins and superaquaporins [7]. Aquaporins are found to mainly function as molecular transporters and involve in many disease progresses such as various chronic kidney disease, systemic disease and cancers [7-10]. Recent studies suggest that Aquaporins could be a promising drug target [11,12].

The human AQP5 gene encodes a protein of 265 amino acids containing six transmembrane domain and five connecting loops [13]. Previous studies indicated that AQP5 protein localized on the membrane of different kinds of cells such as corneal epithelium cells, acinar cells in the lacrimal gland and pulmonary epithelial cells [14]. Overexpression of AQP5 have been reported to be associated with many kinds of cancers such as colorectal, cervical, lung, breast and epithelial ovarian cancer, and to be a potential prognostic biomarkers [15-19]. The role of AQP5 in carcinogenesis is not yet fully understood. Many studies demonstrated that overexpression of AQP5 result in the increase of proliferation, migration and invasion of many kinds of human cancer cell lines [16,20-23]. Phosphorylation of AQP5 by protein kinase A (PKA) has been identified to influence the Ras activity which in turn regulated the proliferation and transformation of NIH3T3 cells [24]. Besides, the NF-kappaB signaling pathway was also proved to have a positive correlation with AQP5 expression in CAOV3 cells [25]. Our previous studies have shown that the expression of AQP5 in malignant and borderline epithelial ovarian tumors is significantly higher than that in benign tumors and normal ovarian tissue [26]. The role of AQP5 in epithelial ovarian tumorigenesis has not previously been clearly evaluated. Therefore, in this study, we used lentivirus mediated RNA interference method to down-regulate the expression to investigate the possible function of AQP5 in epithelial ovarian cancer cells.

## Materials and methods

### Animals

The 6–8 weeks old female BALB/c nude mice purchased from Shanghai Laboratory Animal Center, CAS serves were used to generate the tumor xenografts model. All animals were used and cared for following the Animal Research Advisory Committee of the Shanghai Institute for Biological Sciences.

### Generation of shRNA plasmids

The target sequences against human AQP5 mRNA (GeneBank ID: NM_001651.3) were designed by Invitrogen Block-iT RNAi Designer, a RNAi design software of Invitrogen Corporation. The nucleotide sequences of four candidates were shown in the Table 1. The oligonucleotides were synthesized by Invitrogen Corporation. The annealed nucleotides were then ligated into the linearized pcDNA6.2-GW/EmGFP-miR vector (Invitrogen). All constructs were sequenced for verifying.

**Table 1:** **Sequence of oligonucleotides used in this study**

**Oligonucleotides**	**Sequence**
shRNA-NC-F	5′-TGCTGAAATGTACTGCGCGTGGAGACGTTTTGGCCACTGACTGACGTCTCCACGCAGTACATT-3′
shRNA-NC-R	5′-CCTGAAATGTACTGCGTGGAGACGTCAGTCAGTGGCCAAAACGTCTCCACGCGCAGTACATTTC-3′
shRNA-1#-F	5′-TGCTGCAGTGAAGTAGATTCCGACAAGTTTTGGCCACTGACTGACTTGTCGGACTACTTCACTG-3′
shRNA-1#-R	5′-CCTGCAGTGAAGTAGTCCGACAAGTCAGTCAGTGGCCAAAACTTGTCGGAATCTACTTCACTGC-3′
shRNA-2#-F	5′-TGCTGAACCGATTCATGACCACCGCAGTTTTGGCCACTGACTGACTGCGGTGGATGAATCGGTT-3′
shRNA-2#-R	5′-CCTGAACCGATTCATCCACCGCAGTCAGTCAGTGGCCAAAACTGCGGTGGTCATGAATCGGTTC-3′
shRNA-3#-F	5′-TGCTGATTGAGCGGTGCCACACCGTAGTTTTGGCCACTGACTGACTACGGTGTCACCGCTCAAT-3′
shRNA-3#-R	5′-CCTGATTGAGCGGTGACACCGTAGTCAGTCAGTGGCCAAAACTACGGTGTGGCACCGCTCAATC-3′
shRNA-3#-F	5′-TGCTGATCAGCTCCACCACCATGGCCGTTTTGGCCACTGACTGACGGCCATGGGTGGAGCTGAT-3′
shRNA-3#-R	5′-CCTGATCAGCTCCACCCATGGCCGTCAGTCAGTGGCCAAAACGGCCATGGTGGTGGAGCTGATC-3’

### Cell cultures and transfection

The human epithelial ovarian cancer 3AO cells and human embryonic kidney (HEK) 293 T cells were obtained from the Cell Bank of the Chinese Academy of Sciences (Shanghai, China) and maintained in Dulbecco modified Eagle’s medium (DMEM, Gibco) containing 10% fetal bovine serum (FBS, Gibco) and antibiotics at 37°C with 5% CO_2_. Plasmids were transfected into HEK293T cells and 3AO cells using Lipofectamine 2000 reagent (Invitrogen) according the manufacturer’s protocol for virus package and knock-down efficiency test, respectively.

### Reverse-transcription PCR and qPCR

To verify the knock down efficiency of AQP5 shRNA, 3AO cells were treated with Trizol reagent (Invitrogen) to extract total RNA. RNA was then reverse transcribed by M-MLV transcriptase (Invitrogen) following the manufacturer’s protocol and amplified using SYBR Ex Taq premix (Takara, Japan) by iQ5 multicolor real-time PCR detection system (Bio-Rad). Primers used for qPCR were as follows: human beta-actin (ACTB) (forward primer: 5′- TCCTTCCTGGGCATGGAGT -3′; reverse primer: 5′- CAGGAGGAGCAATGATCTTGAT -3′); human AQP5 (forward primer: 5′- CTGTCCATTGGCCTGTCTGTC -3′; reverse primer: 5′- GGCTCATACGTGCCTTTGATG -3′).

### Western blotting

Lysates from 3AO cells and tissues were prepared in ice-cold RIPA buffer (Beyotime) supplied with protease inhibitor cocktail (Roche) and 1 mM PMSF (Calbiochem). After centrifuge at 13,000 × g for 10 min, the supernatant of lysates was denatured, loaded into the wells of the 12% sodium dodecyl sulfate polyacrylamide gel electrophoresis (SDS-PAGE) gel. The proteins were separated by electrophoresis, transferred to nitrocellulose membranes (Millipore), blocked with 5% nonfast milk in Tris buffered saline (TBS) buffer with 0.05% Tween-20 for 1 hour at room temperature, and then probed with mouse anti-AQP5 antibody (1:1300, Abcam, UK) at 4°C overnight. After incubation for 2 hours with horseradish-peroxidase (HRP)-conjugated rabbit anti-mouse secondary antibody (1:5000, Santa Cruz Biotechnology, USA), bands were visualized by ECL plus western blot detection reagent (GE, USA). The house-keeping gene GAPDH was used as a loading control and detected by using mouse anti-GAPDH antibody (1:4000, abmart, China) plus HRP-conjugated goat anti-mouse secondary antibody (1:5000, Santa Cruz Biotechnology, USA).

### Construction of AQP5 shRNA lentivirus vector and cell infection

The most effective AQP5 shRNA tested by qPCR and western blotting was inserted into plent6.3/V5-Dest vector (Invitrogen) respectively to generate the shRNA lentivirus vector by BP recombination following the standard protocol. The NC control lentivirus vector was constructed in the same way. The generated plasmids were confirmed by sequence and then named as Lenti-AQP5-RNAi and Lenti-NC, respectively. The lentiviral vectors and packaging vectors were then transfected into 293 T cells. Supernatants containing lentivirus expressing AQP5 shRNA or control shRNA were harvested 72 h after transfection and then concentrated by ultracentrifugation to purify lentivirus. The titer of lentivirus was then determined by infected 293 cells. The 3AO cells were infected with lentivirus at multiplicity of infection (MOI) of 30 plus 5 ug/ml Polybrene. The knockdown efficiency of AQP5 by infection was tested by qPCR and western blotting. Then infected cells were used for CCK8 assay and migration assay.

### CCK8 assay

3AO cells infected by AQP5 shRNA lentivirus or control virus were trypsinized, resuspended. Then 5000 cells seeded into 96-well plate for each well. Cell viability was evaluated with Cell Counting Kit-8 (Beyotime) according to the protocol of the manufacture at daily intervals from the next day to the sixth day after seeding. After treated with CCK8 at 37°C for 1 hour, 3AO cells was used to measure the absorbency at 450 nm using a microplate reader.

### Cell migration assay

The migration ability of 3AO cells infected for 3 days was determined using a Transwell chamber (Corning). Briefly, cells were trypsinized and then resuspended by DMEM medium without FBS to the concentration of 2 × 10^6^/ml. The transwell plates (8.0 um pore, Corning Costar) containing 100 ul cell suspension in the upper chamber and 650 ul DMEM medium with 10% FBS in the lower chamber was incubated at 37°C in 5% CO_2_ for 48 h. Then, after removing the cells remaining on the upper surface of the filter, those cells invaded to the lower compartment were fixed with Methanol and stained with crystal violet (GenMed). Cell images were captured in 3 random fields under light microscope at 4 ×, 10 × and 20 × magnification. In addition, invaded cells were segregated, lysed and quantified at 570 nm using spectrophotometer to evaluate the amount of migrated cells.

### Tumor xenografts

Twenty BALB/c nude mice were divided randomly into four groups for tumor Xenografts as mock group, PBS group, Lenti-NC group and Lenti-AQP5-RNAi group. 3AO cells which had been trypsinized and then suspended with DMEM medium were injected subcutaneously into the BALB/c nude mice at a dose of 4 × 10^6^/200 ul for the PBS group, Lenti-NC group and Lenti-AQP5-RNAi group. 200ul DMEM medium without cells were used as a mock control. After tumors were visible in the groups injected with cells, tumor length (L) and width (W) were measured every 3 days and the first day of measurement was marked as day 0. Tumor volume (V) was determined using the simplified formula: V = LW^2^/2. Once the average diameter of tumors researched approximately 4 to 6 mm, the mice received an injection with 50 ul solution into the tumor in multi-position every five days for four times. The PBS buffer, Lenti-NC virus solution and Lenti-AQP5-RNAi virus solution were used for PBS group and mock group, Lenti-NC group and Lenti-AQP5-RNAi group, respectively. Tumors were harvested five days after the fourth treatment.

### Statistical analysis

Statistical analysis was performed by Prism 5.0 software. Data are presented as mean ± SEM. Significance of differences were determined by unpaired, two-tailed Student’s *t* test. P values < 0.05 were considered significant.

## Results

### AQP5 shRNA effectively knock down AQP5 expression

**Figure 1:**
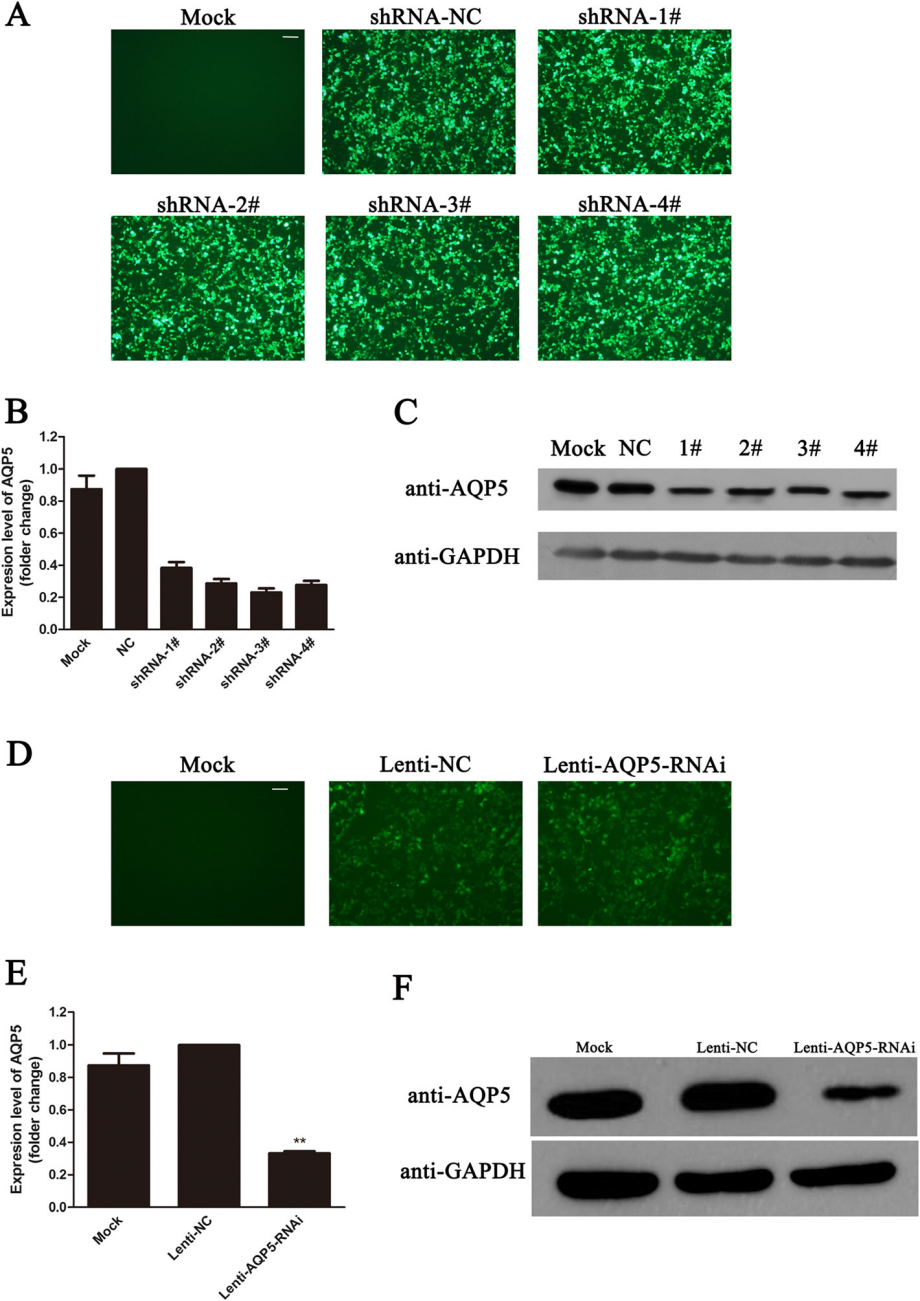
**Knock-down efficiency of the AQP5 shRNA constructs. (A)** Representative images of 3AO cells transfected with indicated plasmids; scale bar, 50 μm. Levels of AQP5 mRNA **(B)** and protein **(C)** in 3AO cells transfected with indicated shRNA contrasts. **(D)** Representative images of 3AO cells infected with indicated lentivirus; scale bar, 50 μm. Expression levels of AQP5 of 3AO cells treated with indicated virus determined by qPCR **(E)** and western blotting **(F)**.

In order to select the most effective shRNA of AQP5, four short-hairpin RNA (shRNA) constructs that target human AQP5 mRNA and a non targeting control shRNA was transfected into 3AO cells, respectively. Images of transfected cell showed these constructs could be transfected into 3AO cells with high efficiency (Figure 1A). Both semiquantitative RT-PCR and western blotting suggested that shRNA-3# was more effective than other shRNAs at knocking down AQP5 (Figure 1B and C).

The shRNA-3# was then inserted into lentivirus vector which was used for lentiviral packaging. The concentrated lentivirus solution was used to infect 3AO cells. Images of infected cell suggested that 3AO cells could be efficiently infected by lentivirus (Figure 1D). The knocking down efficiency of Lenti-AQP5-RNAi lentivirus was proved by both semiquantitative RT-PCR and western blotting (Figure 1E and F).

### Down-regulation of AQP5 decrease cell proliferation of 3AO cells

**Figure 2:**
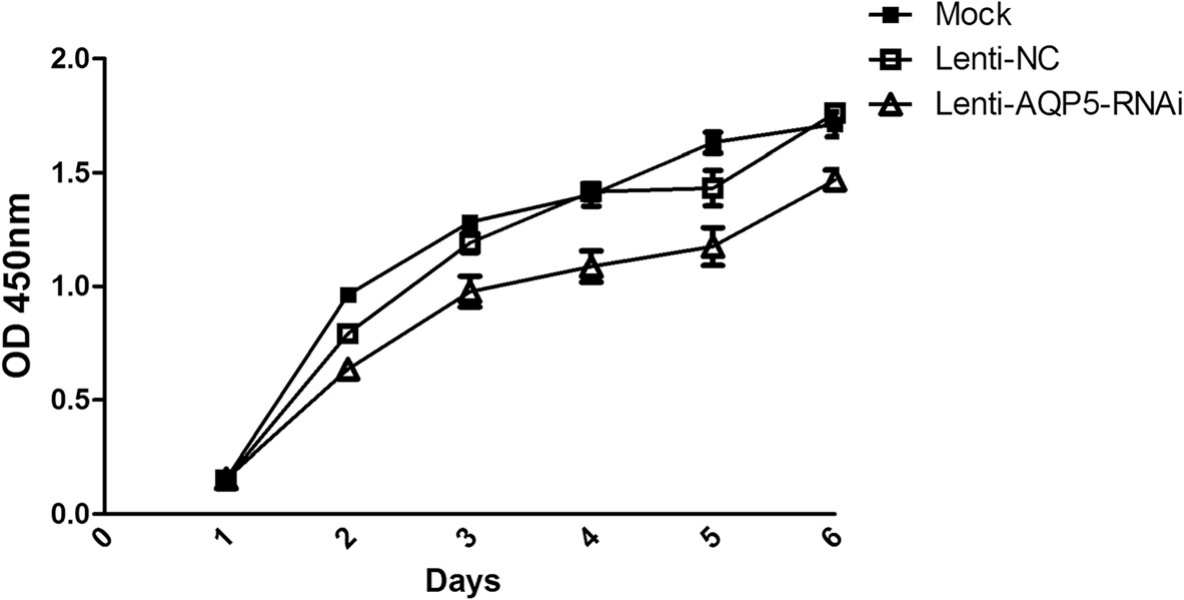
**Knocking-down AQP5 decreases 3AO cells proliferation.** 3AO cells infected with AQP5 shRNA lentivirus and various controls. The CCK8 assay demonstrated that cells treated with AQP5 shRNA have lower viability compared with the controls. The experiment was repeated for three times. Statistic significant differences were observed between cells infected with AQP5 shRNA virus and NC/mock cells.

Our previous worked demonstrated that the high expression AQP5 was correlated with epithelial ovarian tumorigenesis [26]. To investigate the role of AQP5 in ovarian tumorigenesis, we firstly examined whether AQP5 involved in the proliferation of ovarian cancer cells. Cells infected with lentivirus or without treatment were used to measure the cell viability by CCK8 assay for 6 days. Compared to cells without treatment or transfected with Lenti-NC virus, cells with low expression level of AQP5 had a lowed cell proliferation ability (Figure 2). This initial result suggested that AQP5 played a potential role in ovarian cell proliferation.

### APQ5 is involved in cell migration

**Figure 3:**
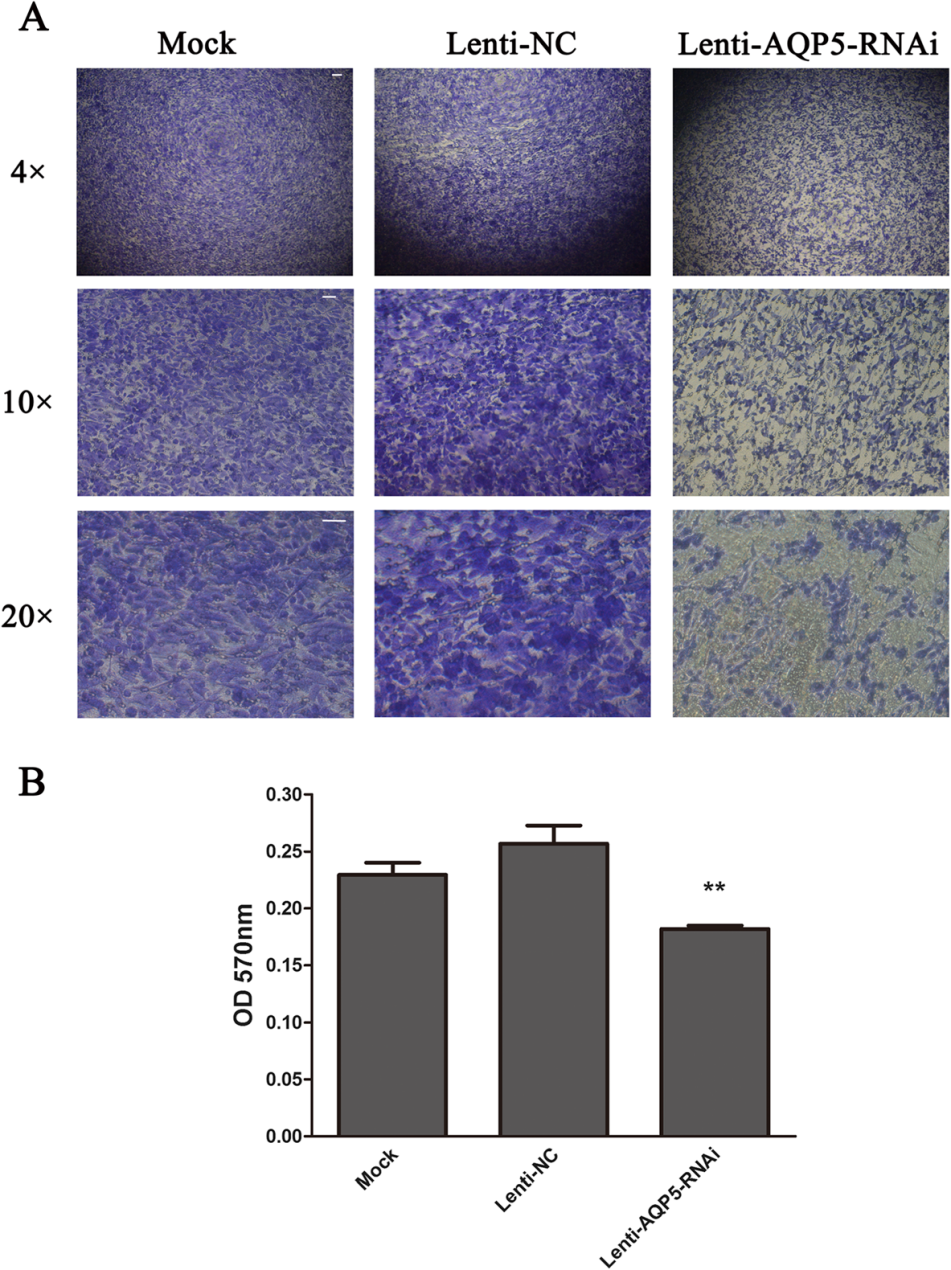
**Knocking-down AQP5 inhibits 3AO cells migration. (A)** Representative images of 3AO cells with indicated treatments; scale bar, 100 μm (4 ×), 50 μm (10 ×) and 50 μm (20 ×). **(B)** Migration ability of 3AO with different infections was analyzed by transwell assay. The experiment was performed for three times. Statistic significant differences were noted between cells infected with AQP5 shRNA virus and NC/mock cells (*t* test, p < 0.01).

To further explore the role of AQP5 in ovarian tumorigenesis, we used Tanswell assay to investigate whether AQP5 is associate with migration ovarian cell. The Images of cells stained with crystal violet suggested that knocking down the expression of AQP5 resulted in the decrease of amount of invaded cells (Figure 3A). Additional observation of absorption assay also proved that the migration ability of 3AO cell reduced after knocking down the expression of AQP5 (Figure 3B). All of these data suggested that AQP5 was involved in the migration of ovarian cancer cells.

### Reduction expression of AQP5 inhibited tumorigenesis in tumor xenografts model

**Figure 4:**
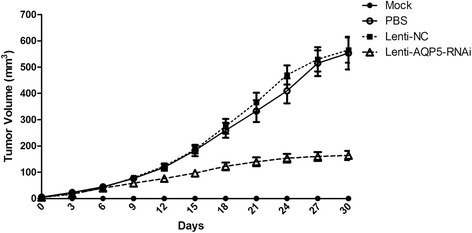
**Down-regulating AQP5 expression appears to inhibit tumor growth in athymic mice.** Tumor growth was observed within 30 days since visible tumor observed. Lentivirus solution or PBS buffer were subcutaneously injected when the average diameter of tumors researched approximately 4 to 6 mm. No tumor growth was observed in mice injected with PBS instead of 3AO cells. Means and standard deviations of tumor volume were calculated from multiple observations in three groups of mice. Statistically significant differences were noted between mice received Lenti-AQP5-shRNA virus injection and mice injected with Lenti-NC virus or PBS buffer (*t* test, p < 0.01). n = 5 per group.

To verify the role of AQP5 in tumorigenesis, we performed xenoplantation in athymic mice with different kinds of injection in the tumorigenesis process. The growth rate of tumor size in mice receiving Lenti-AQP5-RNAi virus started to slow down after virus treatment. While the tumor in other two treatment groups continued to grow rapidly. In contrast, mice in the mock group treated with PBS buffer though the experiment did not form any detectable tumor (Figure 4).

## Discussion

Our previous studies have identified that the expression level of AQP5 in malignant and borderline ovarian tumors is significant higher than those in benign tumors [26]. Our studies also suggested the probable relationship between NF-kappaB pathway and AQP5 expression in human ovarian cancer cells [25]. Our present report gave us more insights into the role of AQP5 in the ovarian carcinogenesis process. It suggested that AQP5 was positively associated with both proliferation and migration of ovarian cancer cells, which may also have affected the growth of tumor in the xenoplantation model. All of these preliminary findings explored the role AQP5 in ovarian cancer and indicated that AQP5 is a putative oncogene and may play multi roles in ovarian carcinoma.

The literature suggested that AQP5 paly a significant role in the cancer process and be a potential early biomarker for many kinds of tumors [2,18,27,28]. While the detailed function of AQP5 in cancer is still unclear. Previous studies proved that deletion of cAMP-protein kinase (PKA) phosphorylation substrate sequence of AQP5 could inhibit the cell proliferative ability of AQP5 in NIH 3 T3 cells. In addition, the PKA consensus site of AQP5 was found to be phosphorylated preferentially in tumors [20]. The following works further demonstrated that the phosphorylation of AQP5 by PKA activated Ras activity which regulated proliferation and transformation of cancer cells [24]. Thus, it is possible that a decrease of AQP5 lead to the inhibition of Ras activity and then cell proliferation which could be investigated in our ongoing studies.

A recent discovery of the involvement of AQP5 in cell migration suggested that AQP5 may determine cell migration ability by mediating water permeability and regulating cell shape and volume [29]. Besides, the upregulation of AQP5 by E2 could increase annexin-2, a regulator of F-actin remodeling and rearrangement, and result in the increase of cell migration, invasion and adhesion in Ishikawa cells [23]. These studies helped us to understand the role of AQP5 in the cancer cell migration process. However, more direct and detailed mechanisms are needed in order to approve the work.

Inhibition of tumor growth by down regulation was also observed in tumor xenografts model. As injection of virus with shRNA target AQP5 was performed after visible tumors were generated, our observation suggested that AQP5 should play an important role in the ovarian carcinogenesis and be a potential drug target for ovarian treatment.

## Conclusions

In conclusion, this report provides systemic evidence of the multi role of AQP5 in ovarian tumorigenesis and indicates that AQP5 is a promising treatment target beyond a biomarker for ovarian prognosis. Further investigations on the pathways APQ5 may involved in are need to understand detailed roles in tumorigenesis.
